# The Effects of COVID-19 Home Confinement in Dementia Care: Physical and Cognitive Decline, Severe Neuropsychiatric Symptoms and Increased Caregiving Burden

**DOI:** 10.1177/1533317520976720

**Published:** 2020-12-09

**Authors:** Flávia Borges-Machado, Duarte Barros, Óscar Ribeiro, Joana Carvalho

**Affiliations:** 1 CIAFEL, Centro de Investigação em Atividade Física, Saúde e Lazer, Universidade do Porto, Porto, Portugal; 2 Departamento de Educação e Psicologia, CINTESIS, Centro de Investigação em Tecnologias e Serviços de Saúde, Universidade de Aveiro, Aveiro, Portugal; 3 Faculdade de Desporto da Universidade do Porto, Porto, Portugal

**Keywords:** dementia, burden, caregiver stress, informal caregiver, SARS-CoV-2

## Abstract

**Purpose:**

This study aims to analyze home confinement impact on individuals with neurocognitive disorders (NCD) through informal caregiver’s perspective and examine how it has affected caregiving burden.

**Methods:**

Thirty-six caregivers (64.94 ± 13.54 years, 41.7% female) of individuals with NCD (74.28 ± 6.76 years, 66.7% female) selected from the *Body & Brain* exercise program were interviewed over the phone. The following instruments were used: Barthel Index (BI) to assess care recipients’ ability to function independently on activities of daily living (ADL), the Neuropsychiatric Inventory (NPI) to evaluate neuropsychiatric symptoms, and the CarerQol-7D/ CarerQol-VAS to determine caregiver subjective burden/well-being.

**Results:**

Pre and post-confinement comparisons showed that care recipients significantly declined their independence in ADL (p = 0.003) and increased NPI total score (MD = 5.72; 95% CI: 1.19 to 10.25, p = 0.015). As for caregivers, results also showed an increased caregiving burden (MD = −0.17; 95% CI: −0.27 to −0.08; p = 0.001) and a decline in their well-being (p = 0.015).

**Discussion:**

COVID-19 crisis sheds light on how imperative it is to find solutions and design contingency plans for future crisis, in order to ensure properly sustained support to dementia caregiving dyads and mitigate caregivers’ burden.

## Introduction

The World Health Organization declared COVID-19 a pandemic. Despite the massive investigation, no effective pharmacological drug or vaccine has been approved to treat or prevent this highly infectious disease.^1,2^ In Portugal, the first cases were confirmed in March 2020 and rapidly the government declared the state of emergency to slow the spread of the virus.^3^ Several draconian measures of physical-social distancing were set to restrict movement and gatherings.^4^ Notwithstanding the effectiveness of these measures, several consequences in the general population have been reported such as increased risk of functional decline, disability and negative psychological effects (e.g. anxiety and depression).^5-7^

Particularly vulnerable groups like older adults with chronic health conditions were asked to remain at home, sometimes imposing their family members with a full time task of providing care without the necessary abilities.^8^ Either by sharing the same household, or by providing support within the necessary distance precaution measures, caregivers, namely children, faced the challenges of working from home, home-schooling children in quarantine, and guaranteeing the instrumental and emotional need of their parents.^9,10^

Individuals diagnosed with a neurocognitive disorder (NCD) might not understand what is happening or be able to follow the necessary safeguard procedures and care for themselves.^11,12^ Indeed, COVID-19 home confinement may have a great impact through the interruption of sustained routines, increasing anxiety and boringness due to lack of activities, conflicts with caregivers, disorganized daily life, cognitive decline, sleep disturbances, and excessive stress and tension.^13,14^ This snowball effect may lead to negative impacts on behavioral and psychological symptoms (BPSD) related to NCD (e.g., delusions, agitation, aberrant motor behavior, intrusiveness or wandering), contributing to even higher levels of stress within the family.^8,15^ While staying at home, older adults are also prone to increase the time spent in sedentary activities and reduce daily physical activity; altogether, these home confinement effects might impact physical function and independence in ADL.^16,17^

Caregiving per se is associated with negative mental health consequences such as distress, depression and anxiety.^18^ As a result of this pandemic, some caregivers might get infected or become ill, which leads to isolation and unavailability to provide care, development of mental health issues, or financial constraints.^8^ Along with the irregularity or lack of outside care assistance, which adds more strain to already-highly levels of burden,^19^ the ceasing of face-to-face activities, such as support groups, may further affect the caregivers’ ability to cope with increased levels of stress or anxiety.^20^

In short, it is imperative to understand and determine the negative effects of this pandemic on caregivers and to coordinate research efforts to improve their quality of life during these particularly challenging times.^9,21,22^ To our knowledge, little is yet known about the effect of home confinement on people with NCD^23-25^ or its impact on caregivers’ burden measured through standardized tests. This study aims to investigate how home confinement impacted care provision of individuals diagnosed with NCD through informal caregivers’ perspective, namely how it has affected the care recipient’s status and caregiving burden.

## Design and Methods

This study is a part of the ongoing project entitled *Body & Brain* (https://ClinicalTrials.gov—identifier number NCT04095962). All participants had already signed an informed consent form and all procedures were conducted in full accordance with Helsinki Declaration. The study protocol was approved by the Ethical Commission of the Faculty of Sports of the University of Porto (CEFADE22.2018). This quasi-experimental controlled trial aims to investigate the effect of a 6-month Multicomponent Training (MT) exercise intervention on the physical and cognitive function of people with neurocognitive disorder. Additionally, the study explores the effect of the MT program on underlying mechanisms associated with NCD (i.e., functional independence in ADL, body composition, quality of life, neuropsychiatric symptoms, caregiver’s burden, blood-based biomarkers and hemodynamics, dietary intake and hydration status). It included individuals aged ≥ 60, capable of walking autonomously without an assistive device or human assistance, and diagnosed with dementia/neurocognitive disorder using accepted diagnostic criteria such as established by the Diagnostic and Statistical Manual of Mental Disorders (DSM-IV-TR or DSM-5),^26^ ICD-10^27^, or the NINCDS-ADRDA.^28^ This multicenter intervention was being conducted in a wide-ranging number of public and private settings (e.g., daycare centers, nursing homes, hospital centers) in the metropolitan area of Porto, Portugal.

This year’s study intervention cycle started in November 2019 but due to COVID-19 pandemic unexpectedly finished on March 6th; afterward, the country declared a state of emergency and all activities ceased. A contingency plan for the project was developed, assuring regular telephone contacts with participants to find out about their overall status and needs.

### Participants

This specific study sample comprises caregivers of participants from the *Body & Brain* project, with a baseline assessment prior to the lockdown period. Only caregivers whose care recipients lived in a nursing home were excluded. Additionally, if the care recipient became severely ill or suffered an acute health event during home confinement period, his/her caregiver was also excluded.

### Data Collection

All evaluations were performed with care recipients’ main informal caregivers who were contacted twice by telephone. First, to collect information about their current status and determine their availability to participate in this specific study, and if they were willing to provide complimentary information on their caregiving situation since COVID-19 home confinement (i.e., routines, number of daily caregiving hours); second, to collect information about the care recipients’ status (independence in ADL, neuropsychiatric symptoms and physical/cognitive status). Finally, participants were asked to provide information about their care management regarding COVID-19 and burden levels. After 5 missed calls, or unavailability to schedule a telephone interview, individuals were excluded.

Baseline data was collected 4 months before the first infected case in Portugal, specifically during November 2019 within the scope of the *Body & Brain* project; and 3 months after home confinement, during June 2020. Results from post-confinement assessments were compared with those of the first evaluations, and specific questions regarding caregivers’ perceptions about care recipients’ behavior were asked to confirm if potential alterations could be attributed to home confinement.

### Care Recipient Evaluation

#### Physical health

Caregivers’ perceptions about changes in physical activity and physical function during home confinement were analyzed through short-answer questions (i.e., comparisons between confinement period with pre-confinement period on the volume of physical activity and sitting time). Caregivers were invited to discuss possible declines and indicate their perception on the physical fitness of the care recipient (e.g., have you noticed differences on the physical fitness of your care recipient, as increased difficulty standing up from a chair or climbing stairs, or gait changes due to home confinement?), and report any fall during this period. Answers were dichotomous: “yes” or “no.”

#### Independence in ADL

Care recipients’ ability to function independently in ADL was assessed with the Barthel Index (BI).^29^ This instrument addresses 10 basic daily activities, such as bathing, dressing or using toilet—with total score ranging from zero to 100. Lower scores indicate higher dependency levels.

#### Neuropsychiatric symptoms

The Neuropsychiatric Inventory (NPI)^30^ was used to analyze the BPSD. The NPI total score is calculated by multiplying the frequency and severity rates per domain (e.g., delusions, agitation, or motor disturbances) and adding them up—ranging from zero to 144 points. Additionally, caregivers were asked to give their perception about care recipients’ alterations over neuropsychiatric symptoms during home confinement.

#### Drug treatment and cognitive function

A short-answer dichotomous question regarding care recipients’ cognitive function alterations during the home confinement was asked to caregivers (e.g., since the beginning of the home confinement, have you noticed a decline in your care recipient’s cognitive function, such as increased difficulties on spatiotemporal orientation or memory problems?). Caregivers were also asked about care recipients’ medication and possible adjustments during home confinement (i.e., dosage changes of ongoing therapies, addition or withdrawal of pharmacological treatment).

### Caregiver Evaluations

#### Caregivers burden

The Care-related Quality of Life instrument (CarerQol) was used to address subjective burden (CarerQol-7D), and caregivers’ well-being (CarerQol-VAS).^31,32^ Subjective burden was measured in 7 dimensions: fulfillment, relational problems, mental health, daily activities problems, physical health and support; caregivers’ well-being was measured in terms of happiness using a visual analogue scale between 0 (completely unhappy) and 10 (completely happy). CarerQol-7D total score ranges from zero to 14, but studies frequently consider mean and standard deviation (SD) values, with higher scores indicating a better caregiving situation.

#### Care management

Caregivers were asked 10 dichotomic questions about the care situation during COVID-19 pandemic and subsequent home confinement. These questions were planned considering other similar studies for older adults,^33^ particularly those diagnosed with NCD.^9,25^ Caregivers were asked about their ability to cope with challenging BPSD, or difficulty to obtain essentials (i.e., food, medication) and opportunity to care for themselves. Each statement required a “yes” or “no” answer, according to the caregiver status—e.g. I felt that proving care during home confinement was physically, emotionally or financially harder than previously to COVID-19 outbreak; I believe that during home confinement I had more conflicts with my care recipient; I felt that during this confinement period I had increased care-related responsibilities; and I perceived that during home confinement I did not have the opportunity to dedicate time for self-care or to rest.

### Statistical Analysis

Descriptive statistics were expressed as mean (SD) for continuous variables and as frequency and percentages for categorical variables. Normal distribution was analyzed by Shapiro-Wilk test. Statistical comparisons between pre and post confinement means were made using paired-samples Student’s t-tests or paired-samples signed test. The comparison between categorical variables was performed using the chi-squared test. Statistical analyses were conducted with SPSS IBM Statistical Software version 25.0 (SPSS, Inc., Chicago, IL) for Windows with a significance level of p < 0.05.

## Results

### Caregivers’ and Care Recipients’ Characteristics

**Figure 1.**
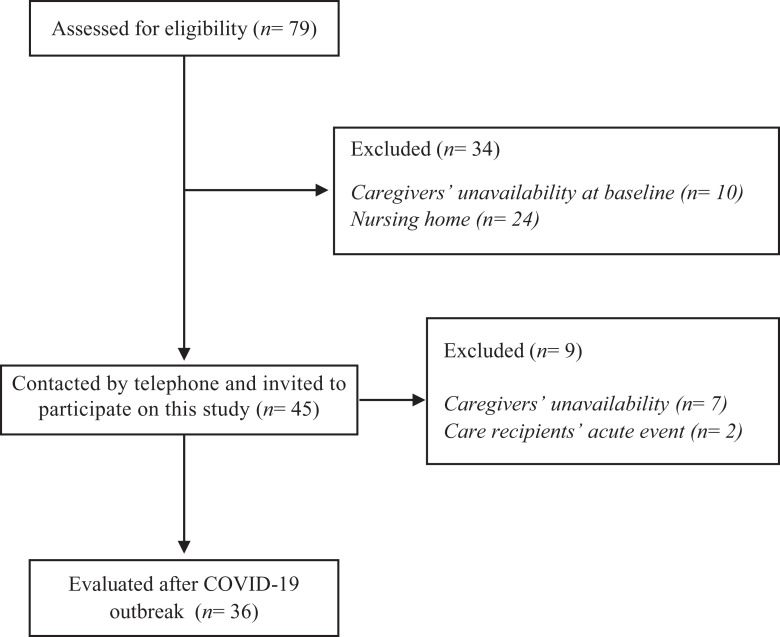
Sample recruitment for this study.

From 79 eligible participants from the ongoing project, 43 were excluded (see study Flowchart). Of those, 2 individuals suffered acute health events during home confinement, and 7 caregivers were not available to participate in these follow-up evaluations (Figure 1). The final sample comprised 36 caregivers [64.94 ± 13.54 years, 15 (41.7%) female] and their care recipients [74.28 ± 6.76 years, 24 (66.7%) female]. Care recipients baseline characteristics are depicted in Table 1. Most of them were married 26 (72.2%) and had low to medium education (63.9%). Half of them (n = 18) used to attend a daycare center.

**Table 1.** Care Recipients’ Sociodemographic Characteristics and Clinical Information.

Characteristics	Care recipients (N = 36)
Age (years), mean (SD)	74.28 ± 6.76
Age, range	62-86
Gender (female), N (%)	24 (66.7%)
Weight (kg), mean (SD)	68.35 ± 9.88
BMI (kg/m^2^), mean (SD)	27.57 ± 4.21
Education, n (%)	
Low (1-3 years)	6 (16.7%)
Medium (4-7 years)	17 (47.2)
High (8-15 years)	13 (36.1)
Day care center	18 (50%)
Living alone, N (%)	3 (8.3%)
Number of medications and supplements, mean (SD)	6.97 ± 3.53
Chronic Health Conditions, n (%)	
Diabetes mellitus	6 (16.7%)
Hypertension	23 (63.9%)
Dyslipidemia	24 (66.7%)
Number of falls in past 12 months, n (%)	
None	27 (75%)
>1	9 (25%)
MMSE, mean (SD)	17.33 ± 6.10
Diagnosis, n (%)	
Dementia or Major NCD	31 (86.1%)
Alzheimer’s Disease	17 (54.8%)
Multiple Etiologies	6 (19.4%)
Unspecified	4 (12.9%)
Vascular Disease	2 (6.5%)
Frontotemporal Disease	1 (3.2%)
Korsakoff Syndrome	1 (3.2%)
Mild cognitive impairment or Minor NCD	5 (13.9%)

Table 2 presents caregivers pre-confinement characteristics. Caregivers were highly educated (72.2%), most were professionally retired (58.2%) with a large heterogenous health status. Nearly half of them rated their general health as “poor or fair” (45.7%) and 58.3% were included in the group of people at increased risk for severe illness from COVID-19.

**Table 2.** Caregivers Sociodemographic Characteristics, Clinical Information and Caregiving Situation.

Characteristics	Caregivers (N = 36)
Age (years), mean (SD)	64.94 ± 13.54
Age, range	36-86
Gender (female), N (%)	15 (41.7%)
Education, N (%)	
Medium (4-7 years)	10 (27.8%)
High (8-15 years)	26 (72.2%)
Professional situation	
Non-Employee	1 (2.8%)
Working	14 (38.9%)
Retired	21 (58.3%)
Caregiver relationship	
Partner	22 (61.1%)
Adult Child	10 (27.8%)
Other relative	4 (11.1%)
Sharing the task of providing care	11 (30.6%)
Number of years as a caregiver, mean (SD)	4 ± 2.16
Providing care (hours/day)	
0-3 h	7 (19.4%)
4-7 h	13 (36.1%)
8-10 h	5 (13.9%)
>10 h	11 (30.6%)
General health	
Poor (N, %)	4 (11.4%)
Fair (N, %)	12 (34.3%)
Good (N, %)	11 (31.4%)
Very Good (N, %)	3 (8.6%)
Excellent (N, %)	5 (14.3%)
Number of medications and supplements, range	0-16
Risk person for COVID	
No	15 (41.7%)
Yes	21 (58.3%)

Overall, 11 (30.6%) caregivers shared the task of providing care with another relative (secondary caregiver) and 5 (13.9%) had professional domiciliary care.

### Caregivers’ Perspective on Physical Activity and ADL Independence of Their Care Recipients

Most caregivers (80.6%) expressed that care recipients decreased their volume of physical activity, and conversely increased their sitting time (Table 3). Additionally, 66.7% mentioned that their care recipients presented physical decline attributed due to home confinement; 8 care recipients (22.2%) felt at least once. Compared to the pre-confinement period, care recipients significantly declined their independence in ADL, presenting a lower BI score (p = 0.003).

**Table 3.** Caregiver’s Perception on Changes in Physical Activity and Physical Function Attributed to Home Confinement Period.

	Total sample (N = 36)
Physical health	
Changes in physical activity	
Increase (N, %)	0 (0%)
Identical (N, %)	7 (19.4%)
Reduced (N, %)	29 (80.6%)
Changes in sitting time	
Increase (N, %)	29 (80.6%)
Identical (N, %)	6 (16.6%)
Reduced (N, %)	1 (2.8%)
Falls	
No (N, %)	28 (77.8%)
Yes (N, %)	8 (22.2%)
Physical decline attributed to home confinement	
No (N, %)	12 (33.3%)
Yes (N, %)	24 (66.7%)
Mental health	
Changes in medication	
No (N, %)	33 (88.9%)
Yes (N, %)	4 (11.1%)
Cognitive decline attributed to home confinement	
No (N, %)	8 (22.2%)
Yes (N, %)	28 (77.8%)
Changes in BSPD	
No (N, %)	20 (55.6%)
Yes (N, %)	16 (44.4%)

### Caregivers’ Perspective About Care Recipients’ Neuropsychiatric Symptoms and Cognitive Function

Most caregivers (80%) mentioned that care recipients presented cognitive decline and 44.4% worsened in their BPSD attributed to home confinement (Table 3). Mean difference between pre and post confinement for NPI total score increased (5.72 ± 13.38, p = 0.015) and reached statistical significance (Table 4).

**Table 4.** Pre and Post Confinement Data in ADL Functional Independence, BPSD and Caregiver Burden.

Total sample (N = 36)			
Care recipients	Pre-confinement	Post-confinement	*p-value*
Barthel Index, Mean ± SD	92.92 ± 9.74	88.33 ± 12.82	p = 0.003^a^
	95 (80-100)	100 (85-100)	
Median (IQR)			
NPI, Mean ± SD	17.56 ± 11.12	23.28 ± 16.81	p = 0.015^b^
Median (IQR)	16.5 (8.25-23.75)	20 (11-34.25)	
Caregiver burden			
CarerQoL-7D, Mean ± SD	1.37 ± 0.38	1.19 ± 0.32	p = 0.001^b^
	1.43 (1.14-1.71)	1.14 (1-1.43)	
Median (IQR)			
CarerQoL-Vas, Mean ± SD	6.83 ± 2.02	5.81 ± 2.55	p = 0.015^a^
	7 (5-8)	7 (5-8)	
Median (IQR)			

Note: ^a^Based on paired-samples signed test; ^b^Based on Student’s paired t-test.

SD, standard deviation; IQR, interquartile range; p < 0.005 significance level.

### Caregivers Burden and Care Management

Caregivers burden (Table 4) increased significantly during home confinement. This can be observed in CarerQoL-7D overall score (−0.17 ± 0.28, p = 0.001). Conversely, caregivers’ self-rated well-being decreased (p = 0.015). This coincided with an increment on time spent in providing care. Before home confinement, 11 (30.6%) caregivers spent more than 10h/day in care provision tasks; presently, 24 (66.7%) reported spending more than 10h/day (χ^ 2^ = 31.46, p < 0.001). Additionally, 6 (16.7%) got unemployed and the same number remained in-person work, while 8.3% started working from home. Within these 2 last scenarios, 5 caregivers reported that care provision was particularly difficult due to fear of infecting the care recipients and/or coordinating working at home with children home-schooling.

Care recipients’ worsening status had a negative impact on their caregivers’ life. Overall, 50% felt it was physically, emotionally or financially harder to take care of their relatives, and 69.4% expressed feelings of an increased sense of responsibility during home confinement. Nearly 40% reported less tolerance to conflicts and 33.3% felt difficulties dealing with BPSD. These perceptions are in line with results obtained from CarerQoL-7D individual dimensions. Compared to the pre-confinement period, there was a significant decline on the perceived fulfillment (χ^ 2^ = 12.94, p = 0.012) and support received in caregiving responsibilities (χ^ 2^ = 9.86, p = 0.043).

## Discussion

The results of this study suggest that COVID-19 outbreak seemed to have significantly augmented caregiving subjective burden and decreased well-being, potentially due to worsening of physical and cognitive functions of people living with NCD. Half of the caregivers reported that it was harder to take care of their relatives. Care recipients significantly increased the severity of neuropsychiatric symptoms and declined their ability to function independently in ADL.

Burton et al. (2015)^34^ highlighted the vicious cycle between lower levels of physical activity and the occurrence of falls, fear of falling, or loss of confidence, which may result in decreased functional ability and loss of daily independence. As expected, due to mandatory home confinement, caregivers perceived that the majority of care recipients increased sitting time and reduced habitual physical activity, which may explain the perceived changes over physical function.^16,17,35^ Furthermore, falls may trigger hospital admission and/or institutionalization.^36^ Despite the multifactorial etiology of NCD, sedentary lifestyle, inactivity and low fitness have been described as determinant risk factors for NCD onset and its progression.^37,38^ Regarding ADL, individuals significantly declined their independence levels. According to Forbes et al. (2015), the mitigation of dependence in ADL as a result of NCD progressing is critical for enhancing the quality of life of both older adults with NCD and their caregivers, and may prevent or delay institutionalization.^39^

Nearly 80% of the caregivers perceived declines in the cognitive function of their relatives due to home confinement. These results are in accordance with Canevelli et al. (2020)^25^ study, which evaluated 139 individuals diagnosed with NCD or other milder cognitive disturbances through a telephone survey to assess care recipients’ changes over cognitive function, neuropsychiatric symptoms and functional independence during home confinement. Overall, caregivers reported worsening of cognitive symptoms—particularly in memory and orientation abilities—on one-third of the sample.^25^ Cognitive decline might be explained by social isolation and/or loneliness, drastic changes on activities and routines, lack of stimulating activities, and absence of appropriate clinical follow-up.^12,25,40-42^

Canevelli and colleagues (2020)^25^ also reported worsening or onset of BPSD on more than half of the care recipients—nearly 55%. This outcome is in accordance with our study findings, since 44.4% of the caregivers perceived that their care recipients presented a worsening of BPSD due to home confinement. Pre and post confinement comparisons showed a statistically significant increasement on severity of neuropsychiatric symptoms, which was superior to the minimal clinically meaningful difference of ≥4 points criteria, suggested by Mega et al.^43,44^ It is important to highlight that these changes are not explained by changes over individuals’ pharmacological therapy since only 4 of them reported adjustments on dosages of ongoing drugs or alteration for new pharmacological treatments. Boutoleau-Bretonniere and collaborators (2020)^23^ showed a similar effect of home confinement over neuropsychiatric symptomatology in 38 individuals diagnosed with Alzheimer’s disease. In short, after a mean duration of 27 days of confinement, 10 individuals presented an increased mean score on the severity subscale of the NPI.^23^ Additionally, Lara et al. (2020)^24^ reported that there was a statistically significant increase in the levels of agitation, apathy, and aberrant motor activity on 40 individuals diagnosed with Alzheimer’s disease or mild cognitive impairment after 5 weeks of domiciliary confinement. Neuropsychiatric symptoms in NCD are common, generally become more severe with disease progression,^45^ and they contributes strongly to caregiver’s distress and burden.^46^ Individuals with NCD who manifest severe neuropsychiatric symptomatology are at higher risk of institutionalization.^46^

Changes over CarerQoL-7D score evidenced the increase of caregivers’ subjective burden, potentially due to increments on the amount of time spent in providing care. Our findings are in accordance with Canevelli et al. (2020)^25^ telephone survey study, in which nearly 50% of caregivers reported higher levels of stress and exhaustion when compared to the pre-confinement moment. Overall, in our study caregivers reported less tolerance to conflicts and increased difficulties in dealing with exacerbated BPSD, which impacted negatively their life. Half of the caregivers felt it was harder to provide care during home confinement and reported increased caregiving responsibilities. These outcomes may be explained by the suspension of formal caregivers’ domiciliary assistance, or support from another family member in providing care. In Portugal, day care centers were closed on March 18th, and although the reopening authorization by health authorities occurred on August 15th most of them remained closed. Furthermore, caregivers training/support groups or psychosocial programs were also suspended, as it was also the case of medical appointments and supplementary exams.^40^

As stated by Greenberg et al. (2020)^9^ the home confinement period amplified caregivers tasks as a result of the daily challenges and lifestyle restrictions. Drastic modifications in routines and the closure of services and facilities, normally used by caregivers for respite care, have negatively impacted both caregivers and people with NCD.^9,12^ Social isolation may be particularly deleterious to caregivers, since having a family member with NCD tends to isolate families due to stigma and deterioration of communication skills.^40^ Furthermore, countless hours spent caregiving could precipitate feelings of loneliness and intensify the stress levels of caregivers.^9^

In conclusion, our findings suggest that home confinement was perceived to have negatively impacted individuals with NCD and their caregivers in distinct levels. Psychosocial and mental health support is not only urgently needed for individuals with NCD,^47,48^ but also for their caregivers.^12,22^

Along with confirming emergent findings on the effects of home confinement in NCD caregiving from the caregivers’ perspective, this study has the merit of further presenting such an impact by means of standardized measurements in comparison with pre-confinement available assessments. However, it is important to acknowledge that baseline evaluations were performed 4 months before home confinement while individuals were participating in the *Body & Brain* program activities. Therefore, results must be interpreted with caution. Additionally, the small sample size does not allow these results to be generalized.

## Implications

The COVID-19 health crisis should not overshadow the neurocognitive disorders pandemic. The number of people experiencing cognitive decline may increase, not only due to COVID-19 direct and indirect effects, but also due to the decreased number of diagnosis of NCD being conducted during the outbreak, which will negatively impact people’s prognosis and treatment, and consequently increase difficulties and burden to their family.^40^ It is urgent to create strategies that may help to provide care, improve the well-being and quality of life, and reduce care burden in caregivers during this pandemic situation. This crisis may expedite the development of non-pharmacological interventions that can be delivered at the care recipients’ home, like home-based cognitive training or physical exercise^8^ and other technology-based approaches.^49^ Lastly, as suggested by Greenberg and collaborators (2020),^9^ caregivers may need to reconnect with pre-confinement existent support systems or even find new ones.

Researchers and policymakers must analyze and create strategies to maintain caregivers and people with NCD living at their homes, safely protected from a possible COVID-19 second wave. In short, particularly daycare centers and domiciliary services should define a contingency plan to warrant the continuity of care providing services.
